# Association between *MTHFR* Polymorphisms and Acute Myeloid Leukemia Risk: A Meta-Analysis

**DOI:** 10.1371/journal.pone.0088823

**Published:** 2014-02-20

**Authors:** Yu-Tao Qin, Yong Zhang, Fang Wu, Yan Su, Ge-Ning Lu, Ren-Sheng Wang

**Affiliations:** Department of Radiotherapy, the First Affiliated Hospital of Guangxi Medical University, Nanning, Guangxi, People’s Republic of China; National Cancer Center, Japan

## Abstract

Previous observational studies investigating the association between methylenetetrahydrofolate reductase (*MTHFR*) polymorphisms and acute myeloid leukemia risk (AML) have yielded inconsistent results. The aim of this study is to derive a more precise estimation of the association between *MTHFR* (C677T and A1298C) polymorphisms and acute myeloid leukemia risk. PubMed and Embase databases were systematically searched to identify relevant studies from their inception to August 2013. Odds ratios (ORs) with 95% confidence intervals (CIs) were the metric of choice. Thirteen studies were selected for C677T polymorphism (1838 cases and 5318 controls) and 9 studies (1335 patients and 4295 controls) for A1298C polymorphism. Overall, pooled results showed that C677T polymorphism was not significant associated with AML risk(OR, 0.98–1.04; 95% CI, 0.86–0.92 to 1.09–1.25). Similar results were observed for the A1298C polymorphism and in subgroup analysis. All comparisons revealed no substantial heterogeneity nor did we detect evidence of publication bias. In summary, this meta-analysis provides evidence that *MTHFR* polymorphisms were not associated with AML risk. Further investigations are needed to offer better insight into the role of these polymorphisms in AML carcinogenesis.

## Introduction

Worldwide, an estimated 57 000 cases of leukemia occur every year [1] and acute myeloid leukemia (AML) is the most common acute leukemia (AL). The highest incidence rate is found in males of all age groups, the fact remains to be explained [2]–[4]. The etiology of most types of leukemia is still unknown. Leukemia is likely to be associated with certain environmental agents, such as ionizing radiation, benzene, and cancer chemotherapy. The increase risk factors for leukemia may be both quantity and quality changes in folic acid metabolism [5]–[7].

The folate metabolites of carcinogens can influence the gene expression and DNA instability. DNA translocations, inversions or deletions in haematopoietic progenitor cells will lead to leukemia. Be short of folate can result in a lot of cellular disorders [8], [9]. Folate metabolism participates in processes of DNA methylation, as well as involves in the synthesis and repair of DNA. That is a mechanism to prevent and repair damaged DNA [10]. The 5, 10-methylenetetrahydrofolate reductase (*MTHFR*) gene is found at the end of the short arm of chromosome one at locus 1p36.3. The complementary DNA sequence of this gene is approximately 2.2 kb, made up of 11exons (103–432 bp). The major product of *MTHFR* locus in human is a 77-kilodaltonprotein [11]. *MTHFR* plays a pivotal role in the folate metabolism, it can catalyze the irreversible conversion of 5, 10-methylenetrahydrofolate to 5-methylenetrahydrofolate, which participates in the remethylation of homocysteine to methionine [12]. Two common polymorphisms in *MTHFR*, C677T and A1298C, have been associated with reduced enzyme activity of *MTHFR*, which lead to an accumulation of 5, 10-methylenetetrahydrofolate and DNA hypomethylation. The 5,10-methylenetetrahydrofolate donates a methyl group, which converts dUMP to dTMP and repairs DNA damages [11]. C677T polymorphism occurs in exon4, which leads alanine to be substituted by valine at codon222. People with the homozygous *MTHFR* 677TT genotype have 30 percent enzyme activity compared with those having wild-type allele, while those with heterozygous *MTHFR* 677 CT allele have 60 percent enzyme activity [11]. This polymorphism promotes the separation of enzyme from its co-factor, which results in the enzyme activity decrease [13]. Recently, another important polymorphism in the *MTHFR* gene is A1298C in exon7, which leads to a glutamate-to-alanine (A>C) change and reduced enzyme activity of *MTHFR*.

To date, several studies have investigated the association between *MTHFR* polymorphisms and AML risk [7], [11], [14]–[30], but results from those studies remain inconsistent. Therefore, we conducted a meta-analysis of previously published studies to assess the relationship between the *MTHFR* polymorphisms and AML risk.

## Methods

### Search Strategy and Selection Criteria

Eligible studies were identified by searching electronic literature databases PubMed and Embase (from inception to August 2013). The search strategy used the following keywords: *MTHFR*, polymorphism, acute myeloid leukemia or acute myeloblastic leukemia. We did not apply language restrictions. References of reviews or original studies identified in the literature search were hand searched for additional studies. Studies were included if they met the following inclusion criteria: (1) explored the association of *MTHFR* (C677T and A1298C) polymorphisms with AML risk; (2) used a case-control design; (3) provided available genotype or allele frequency of the cases and control to calculate ORs with 95% CIs. The exclusion criteria also applied: the data from study were repeated or overlapped; there was no available genotype or allele frequency; the patients were about therapy-related AML; the studies were review, case report, or comment.

### Data Extraction

Two investigators (YTQ and FW) independently extracted data using a standardized data-collection form. Study characteristics extracted from each article were as follows: first author, year of publication, country of origin, racial decent, participant age, number of participants, source of controls, genotype studied, and available genotype frequency information for *MTHFR* C677T and A1298C. Any disagreements were resolved by consensus and a third author (YZ). All data were extracted from the published studies and no authors were contacted to require further information.

### Statistical Analysis

The strength of the association between *MTHFR* (C677T and A1298C) polymorphisms and AML risk was measured by using crude odds ratio (OR) with 95% confidence interval (CI). The pooled ORs were estimated in following models: allele contrast (T *vs.* C), codominant model (CT *vs.* CC; TT *vs.* CC), dominant model (TT+CT *vs.* CC), and recessive model (TT *vs.* CT+CC), respectively. For *MTHFR* A1298C polymorphism, we assessed the same association. The Cochran Q test was used to test statistical heterogeneity. The *I* ^2^ statistics [31] was also calculated to quantify the proportion of the variations across studies. A *P* value of less than 0.1 for the Q statistic was considered as heterogeneity across studies, allowing for the use of a random-effects model (DerSimonian and Laird method [32]. Otherwise, a fixed-effects model (Mantel–Haenszel method [33]) was applied. Subgroup analysis based on ethnicity (Caucasian, Asian, and Brazilian), sample size (large sample size ≥100, and small sample size < 100), and HWE was performed to assess the source of heterogeneity. We also assessed the influence of individual studies on the combined risk estimate by sequentially omitting one study each time.

Potential publication bias was assessed both by visually inspecting of the Begg funnel plot and statistically via Egger’s unweighted regression tests [34]. All statistical analyses were conducted using Stata version 11.0 (Stata Corporation, College Station, TX). All *P* values are tailed where 0.05 was considered statistically significant except in the test for heterogeneity.

## Results

### Identification of Eligible Studies

The search strategy yielded 35 potential studies from PubMed and Embase databases. However, most of them were excluded after reviewing titles and abstracts, leaving 19 for full-text review. The literature search and detailed study selection procedures were presented in **Figure S1**. Six studies were excluded (two studies [26], [27] were conference articles, and two [28], [29] with patients were about therapy-related AML, one [11] was review article, and one [30] was supplementary material). Finally, 13 studies [7], [14]–[25] were included in this meta-analysis.

### Study Characteristics

The main characteristics of the included studies were shown in **Table 1**. These studies were published between 1999 and 2012. Sample size ranged from 27 to 1,700 (including 1,838 patients with AML and 5,318 healthy controls). Among these, five studies were in Caucasian descent [17], [19]–[22], five studies of Asian descent [14], [18], [23]–[25] and three studies of Brazilian descent [7], [15], [16]. Thirteen studies including 1838 cases and 5318 controls had examined the association of *MTHFR* C677T polymorphism with AML risk, and 9 studies with a total of 1335 patients and 4295 controls investigated the association between *MTHFR* A1298C polymorphism and AML risk. Of these, 12 studies were population-based and one was hospital-based.

**Table 1:** Characteristics of studies included in this meta-analysis.

First author	Year	Country	Racial decent	Cases, n	Controls,n	Source ofcontrols	HWE	Studied *MTHFR* genotypes
Hussain [14]	2012	India	Asian	112	251	Population	yes	C677T
Lightfoot [19]	2010	United Kingdom	Caucasian	89	824	Population	yes	C677T and A1299C
Vahid [20]	2010	Iran	Caucasian	106	97	Population	yes	C677T and A1299C
Amorim [15]	2008	Brazil	Brazilian	50	248	Population	yes	C677T and A1299C
Kim [24]	2008	Korea	Asian	389	1700	Population	yes	C677T and A1299C
Barbosa [7]	2008	Brazil	Brazilian	27	100	Population	yes	C677T and A1299C
Bolufer [22]	2007	Spain	Caucasian	302	454	Population	yes	C677T
Moon [23]	2007	South Korea	Asian	200	434	Population	yes	C677T and A1299C
Chen [25]	2006	China	Asian	40	157	Population	yes	C677T
Costa Ramos [16]	2006	Brazil	Brazilian	182	315	Population	yes	C677T and A1299C
Hur [18]	2006	Korea	Asian	55	200	Population	no	C677T and A1299C
Deligezer [17]	2003	Turkey	Caucasian	49	161	Population	yes	C677T
Skibola [21]	1999	United Kingdom	Caucasian	237	377	Hospital	yes	C677T and A1299C

HWE, Hardy-Weinberg equilibrium; *MTHFR*, Methylenetetrahydrofolate reductase.

### MTHFR C677T

**Figure 1:**
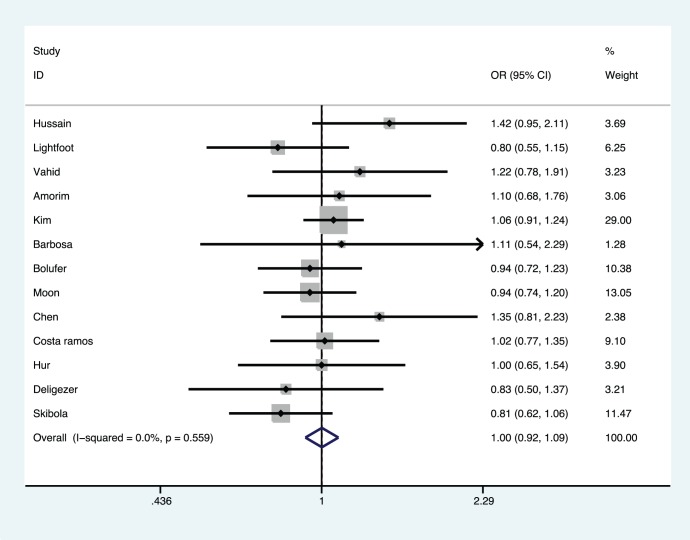
Meta-analysis for the association of acute myeloid leukemia risk with *MTHFR* C677T polymorphism (T *vs.* C).

**Figure 1** showed the results from a fixed-effects model combining the ORs for the association of MTHFR C677T polymorphism and AML risk. Overall, the pooled results showed that the MTHFR C677T polymorphism was not associated with the development of AML (OR, 0.98–1.04; 95% CI, 0.86–0.92 to 1.09–1.25; P, 0.750–0.976), without statistically significant between-study heterogeneity (I^2^, 0.0%–26.4%; P for heterogeneity, 0.178–0.573). Table 2 showed that the Asian and Brazilian subgroups were at increased risk in some genetic models. Caucasians may even have some low-level protection in some models (OR 0.81–0.89).

**Table 2:** Distribution of *MTHFR* C677T genotypes and allelic frequencies in acute myeloid leukemia patients.

Geneticcomparisons	Population andsubgroups under analysis	Studies	Fixed-effects model			
			OR (95% CI)	*p*-value	I2,%	*p* for heterogeneity
T vs. C	All	13	1.00 (0.92–1.09)	0.976	0.0	0.559
	Caucasian	5	0.89 (0.76–1.03)	0.119	0.0	0.573
	Asian	5	1.07 (0.95–1.20)	0.279	0.0	0.417
	Brazilian	3	1.04 (0.83–1.31)	0.720	0.0	0.951
	Large sample size	7	1.01 (0.92–1.11)	0.862	15.4	0.312
	Small sample size	6	0.97 (0.80–1.18)	0.776	0.0	0.629
	All in HWE	12	1.00 (0.92–1.09)	0.976	0.0	0.473
CT vs. CC	All	13	0.98 (0.86–1.11)	0.750	10.5	0.340
	Caucasian	5	0.81 (0.66–1.01)	0.056	26.0	0.248
	Asian	5	1.14 (0.95–1.36)	0.169	0.0	0.680
	Brazilian	3	0.94 (0.69–1.30)	0.722	0.0	0.824
	Large sample size	7	0.99 (0.86–1.14)	0.873	0.0	0.578
	Small sample size	6	0.95 (0.73–1.24)	0.704	42.1	0.125
	All in HWE	12	0.96 (0.84–1.09)	0.530	0.0	0.455
TT vs. CC	All	13	1.04 (0.87–1.25)	0.648	2.9	0.417
	Caucasian	5	0.88 (0.64–1.21)	0.427	0.0	0.411
	Asian	5	1.12 (0.88–1.42)	0.370	41.7	0.143
	Brazilian	3	1.20 (0.72–1.97)	0.484	0.0	0.997
	Large sample size	7	1.05 (0.86–1.29)	0.606	28.8	0.209
	Small sample size	6	1.00 (0.66–1.51)	0.985	0.0	0.553
	All in HWE	12	1.05 (0.88–1.27)	0.570	7.9	0.367
TT+CT vs. CC	All	13	0.99 (0.88–1.12)	0.913	0.0	0.573
	Caucasian	5	0.83 (0.68–1.01)	0.061	0.0	0.433
	Asian	5	1.14 (0.96–1.35)	0.143	0.0	0.933
	Brazilian	3	0.99 (0.74–1.34)	0.965	0.0	0.875
	Large sample size	7	1.00 (0.88–1.15)	0.956	0.0	0.585
	Small sample size	6	0.96 (0.74–1.23)	0.737	12.3	0.336
	All in HWE	12	0.98 (0.87–1.11)	0.762	0.0	0.580
TT vs. CT+CC	All	13	1.02 (0.86–1.20)	0.836	26.4	0.178
	Caucasian	5	0.95 (0.71–1.29)	0.748	15.4	0.316
	Asian	5	1.01 (0.82–1.26)	0.892	63.3	0.028
	Brazilian	3	1.23 (0.76–1.99)	0.398	0.0	0.985
	Large sample size	7	1.02 (0.86–1.23)	0.797	42.2	0.110
	Small sample size	6	0.99 (0.67–1.46)	0.950	16.6	0.306
	All in HWE	12	1.04 (0.88–1.23)	0.631	24.3	0.205

*MTHFR*, methylenetetrahydrofolate reductase; OR, odds ratio; CI, confidence interval; vs., versus; HWE, Hardy-Weinberg equilibrium.

### *MTHFR* A1298C

**Figure 2:**
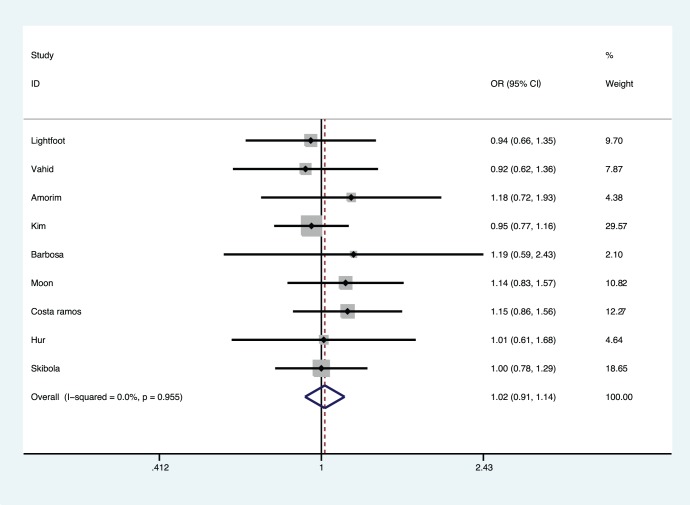
Meta-analysis for the association of acute myeloid leukemia risk with *MTHFR* A1299C polymorphism (C *vs.* A).

Figure 2 presented the results from a fixed-effects model combining the ORs for the association of *MTHFR* A1298C polymorphism and AML risk. Overall, the estimate results indicated non-significant increased risk association of *MTHFR* A1298C polymorphism with AML risk in some genetic models (OR, 1.11–1.13), without zero heterogeneity (*P* for heterogeneity, 0.562–0.955). Table 3 shows that the Brazilian subgroup are at increased risk in all genetic models (OR, 1.1–1.4), and in two genetic models, so are the Asians (OR, 1.23–1.25) as well as the HWE studies (OR, 1.11) and even small sample size studies (OR, 1.36–1.50).

**Table 3:** Distribution of *MTHFR* A1298C genotypes and allelic frequencies in acute myeloid leukemia patients.

Geneticcomparisons	Population andsubgroups under analysis	Studies	Fixed-effects model			
			OR (95% CI)	*p*-value	I2,%	*p* for heterogeneity
C vs. A	All	9	1.02 (0.91–1.14)	0.733	0.0	0.955
	Caucasian	3	0.97 (0.81–1.16)	0.717	0.0	0.926
	Asian	3	1.00 (0.85–1.18)	0.993	0.0	0.625
	Brazilian	3	1.16 (0.91–1.48)	0.216	0.0	0.995
	Large sample size	5	1.02 (0.90–115)	0.808	0.0	0.746
	Small sample size	4	1.03 (0.82–1.31)	0.785	0.0	0.878
	All in HWE	8	1.02 (0.91–1.14)	0.736	0.0	0.916
AC vs. AA	All	9	0.98 (0.85–1.13)	0.760	0.0	0.801
	Caucasian	3	0.97 (0.74–1.26)	0.795	36.7	0.206
	Asian	3	0.95 (0.78–1.15)	0.593	0.0	0.880
	Brazilian	3	1.09 (0.79–1.49)	0.614	0.0	0.723
	Large sample size	5	1.01 (0.87–1.19)	0.857	0.0	0.859
	Small sample size	4	0.84 (0.61–1.16)	0.291	0.0	0.523
	All in HWE	8	0.99 (0.85–1.14)	0.838	0.0	0.732
CC vs. AA	All	9	1.13 (0.86–1.48)	0.378	0.0	0.792
	Caucasian	3	0.97 (0.66–1.42)	0.860	0.0	0.666
	Asian	3	1.23 (0.74–2.02)	0.425	3.7	0.354
	Brazilian	3	1.42 (0.82–2.47)	0.213	0.0	0.847
	Large sample size	5	1.06 (0.78–1.45)	0.715	0.0	0.486
	Small sample size	4	1.36 (0.81–2.28)	0.250	0.0	0.903
	All in HWE	8	1.11 (0.85–1.46)	0.447	0.0	0.744
CC+AC vs. AA	All	9	1.00 (0.88–1.14)	0.995	0.0	0.940
	Caucasian	3	0.96 (0.75–1.23)	0.752	0.0	0.541
	Asian	3	0.97 (0.81–1.17)	0.762	0.0	0.782
	Brazilian	3	1.14 (0.85–1.54)	0.377	0.0	0.895
	Large sample size	5	1.02 (0.88–1.18)	0.796	0.0	0.899
	Small sample size	4	0.93 (0.69–1.25)	0.616	0.0	0.677
	All in HWE	8	1.00 (0.88–1.15)	0.947	0.0	0.900
CC vs. AC+AA	All	9	1.11 (0.86–1.44)	0.415	0.0	0.562
	Caucasian	3	0.95 (0.68–1.38)	0.797	23.4	0.271
	Asian	3	1.25 (0.76–2.06)	0.379	2.9	0.357
	Brazilian	3	1.39 (0.81–2.38)	0.234	0.0	0.762
	Large sample size	5	1.01 (0.75–1.37)	0.939	8.7	0.357
	Small sample size	4	1.50 (0.91–2.48)	0.113	0.0	0.896
	All in HWE	8	1.110 (0.84–1.43)	0.495	0.0	0.508

*MTHFR*, methylenetetrahydrofolate reductase; OR, odds ratio; CI, confidence interval; vs., versus; HWE, Hardy-Weinberg equilibrium.

### Publication Bias

**Figure 3:**
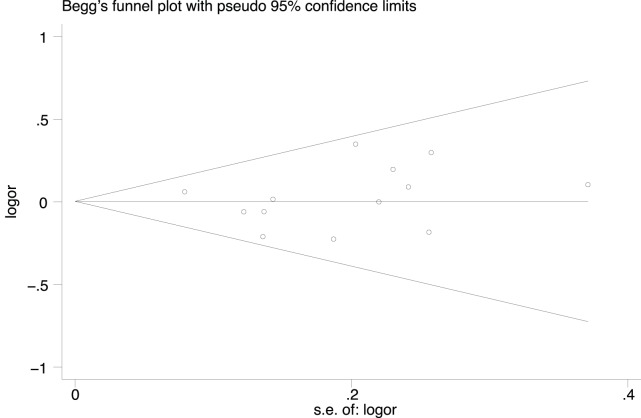
Publication bias test (*MTHFR* C677T: T *vs.* C).

The Begg rank correlation test and Egger linear regression tests for publication bias in the meta-analysis indicated no obvious publication bias among studies (Begg’s test, P = 0.360; Egger’s test, P = 0.659; Figure 3).

## Discussion

To the best of our knowledge, this is the first meta-analysis to assess the association between *MTHFR* polymorphisms and AML risk. Thirteen studies (1838 cases and 5318 controls) and 9 studies (1335 patients and 4295 controls) explored the association between the C677T and A1298C polymorphisms and AML risk, respectively. Results of this study suggested that *MTHFR* (C677T and A1298C) polymorphisms were not significantly associated with AML risk. Moreover, similar results were observed in subgroup analyses based on ethnicity, sample size, and HWE in controls.

Nowadays, several meta-analyses have been performed to clarify the association between *MTHFR* (C677T and A1298C) polymorphisms and risk of several cancers. For instance, You et al have demonstrated that the *MTHFR* C677T and A1298C polymorphisms were associated with bladder cancer risk [35]. Wei et al provided evidence that the *MTHFR* C677T polymorphism increased the risk for developing colorectal cancer [36]. However, a meta-analysis by Ding et al indicated that no significant association was observed between *MTHFR* C677T polymorphism and susceptibility to ovarian cancer [37]. Besides, Niu et al suggested that no significant association between *MTHFR* A1298C polymorphism head and neck cancer [38], which were consistent with our results. These inconsistent and confusing conclusions can be attributed to several factors. Different selection criteria and selection bias might account for the diversity of the results. In addition, the reason might be the complexity of the folate metabolic pathway because MTHFR is only one of many enzymes involved in the pathway. Moreover, the studies with small sample size will have a lower statistical power than those with large sample size. Furthermore, the different mechanisms of carcinogenesis of different cancers might due to gene–variant associations vary in different kinds of diseases.

Several studies have demonstrated that individuals with *MTHFR* 677 TT genotype, lack of vitamins B6 and B12, methionine and folate, and high consumption of alcohol are at increased risk of developing colorectal tumors [39]–[42]. However, no studies have reported these gene-nutrient interactions with the risk of AML. The present study was lack of data to estimate the association of gene-nutrient and risk of AML. These interesting clues may be useful for future research. Dietary intake of several nutrients could influence the distribution of intracellular folate metabolites. Vitamins B6 and B12 may affect DNA synthesis and MTHFR enzyme activity. Moreover, high consumption of alcohol might take place of more nutritious foods, which may lead to the intake deficiency of folate and B vitamins [43]. Deficiency of folate is associated with carcinogenesis mainly in two ways [8]: (1) The conversion of dUMP to dTMP, using for DNA synthesis and repair, demands methyl group donated by 5, 10-methylene*THF*, so lack of folate can intervene thymidylate biosynthesis and then lead to leads to errors in DNA synthesis, strand breakage, and chromosomal repair. (2) Low-level 5-methyl*THF* may result in DNA hypomethylation and cause proto-oncogene expression due to cellular S-adenosylmethionine used up. Thus, cohort studies are needed to focus on gene-nutrient interactions in the future.

In order to better estimate the association of MTHFR (C677T and A1298C) polymorphisms with AML risk, subgroup analysis based on ethnicity, sample size and HWE, was performed. Although Asian and Brazilian subgroups were at increased risk in some genetic models, no significant associations between *MTHFR* (C677T and A1298C) polymorphisms and AML risk were found in sample size subgroups or all in HWE, which indicated that the results of our analysis was reliable and stable. The real effect of *MTHFR* (C677T and A1298C) polymorphisms may be concealed by the causal genes in AML. Moreover, different ethnicity of genotypic milieu and living surroundings might have an effect on AML risk, which may led to an effect in our results.

Several limitations might be acknowledged in this meta-analysis. First, we only selected the published articles to acquire data for analyses, and the unpublished article’s effect was unknown. Thus, it is necessary to conduct a system review to avoid the potential effect in analysis. Second, our study was based on single-factor estimate, which explained the effects of two polymorphisms on AML risk respectively and lack of combination of two polymorphisms analysis. So, conducting a meta-analysis to investigate the combination of these two functional polymorphisms may offer better insight into *MTHFR* (C677T and A1298C) polymorphisms on AML risk. Third, there were no significant effects for both polymorphisms. Fourth, gene-gene and gene-environment interactions might also be considered in future studies. In spite of these, our meta-analysis also has two advantages as follows: (1) there was no significant absence of evidence of publication bias in the present study, which highlighted further, ensured the reliability of association analysis our findings. (2) There was no evidence of statistical heterogeneity between the analyses of two polymorphisms and AML risk underpins the combinability of the component studies.

In conclusion, our meta-analysis indicates that *MTHFR* C677T polymorphism is not associated with AML risk, as well as A1298C polymorphism. Future well-design study is warranted to estimate the effect of combination of two polymorphisms and gene-environment interactions. If epidemiologic study confirms the role of gene-environment interactions, additional studies will be needed to further elucidate the potential biological mechanisms involved.

## Supporting Information

Figure S1 **Flow chart.**
(DOC)

Checklist S1 **PRISMA checklist.**
(DOC)
