# Supplementary material for: Association between MTHFR Polymorphisms and Acute Myeloid Leukemia Risk: A Meta-Analysis
Source: PLoS One. 2014 Feb 20;9(2):e88823. doi: 10.1371/journal.pone.0088823 (PMC3930602; doi:10.1371/journal.pone.0088823)
Supplement: Figure S1 — Flow chart. (DOC) [file pone.0088823.s001.doc]

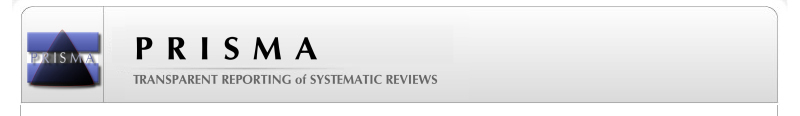
**PRISMA 2009 Flow Diagram**

**Screening**

**Included**

**Eligibility**

**Identification**

Records identified through English database

(PubMed n = 19 Embase n = 16)

Additional records identified through hand-search
(n = 4)

Records after duplicates removed
(n = 39)

Records screened
(n = 30)

Records excluded
(n = 11)

Full-text articles assessed for eligibility
(n = 19)

Full-text articles excluded, with reasons
(n = 6):

1 review

1 supplementary material

2 conference articles

2 therapy-related leukaemia

Studies included in qualitative synthesis
(n = 13)

Studies included in quantitative synthesis (meta-analysis)
(n = 13 )
